# Microbial carbonate reservoir characteristics and their depositional effects, the IV Member of Dengying Formation, Gaoshiti-Moxi area, Sichuan Basin, Southwest China

**DOI:** 10.1038/s41598-023-45044-z

**Published:** 2023-11-03

**Authors:** Yuan Wang, Shaoyong Wang, Haijun Yan, Yijie Zhang, Zhenyu Zhao, Debo Ma

**Affiliations:** PetroChina Research Institute of Petroleum Exploration & Development, Beijing, 100083 China

**Keywords:** Environmental sciences, Solid Earth sciences

## Abstract

Previous scientific research on reservoirs of the Deng IV Member in the Gaoshiti-Moxi area, the main production area of the Anyue gas field with reserves of tens of billions of cubic metres, has focused on karst palaeogeomorphology reconstruction, the facies distribution on platform margins, and their effects on creating favourable reservoirs. However, the quality of microbial carbonate reservoirs is also closely related to their original depositional environments on both inner and marginal platforms. Therefore, this paper attempts to reveal favourable microbial carbonate reservoir characteristics and the sedimentary effects on their distribution and prediction based predominantly on a synthetic analysis of the sequence stratigraphy and depositional facies. The results show that favourable reservoirs of the Deng IV Member are classified into three types according to their reservoir spaces: fracture-cavity, pore-cavity and pore reservoirs. Secondary dissolution pores and cavities are primary reservoir spaces developed mainly in nonskeletal grain dolomites with sparry cements, thrombolites, and stromatolites. The physical properties of the fracture-cavity and pore-cavity types of reservoirs are better than those of pore reservoirs and have porosities between 1 and 5% and permeabilities between 0.01 × 10^–3^ and 1 × 10^–3^ μm^2^. Vertically, favourable reservoirs are developed mainly in parasequence set 6 (PSS6) and PSS7 and are laterally distributed in well zone MX9-MX19-MX1 for the fracture-cavity type, MX105-MX110-GS20 for the pore-cavity type and MX17-MX107-MX41-MX102-GS102 for the pore type. Moreover, depositional effects on reservoirs in terms of depositional sequences, seismic facies, microfacies and microfacies associations indicate that to some extent, the fracture-cavity type of reservoir is constrained by the top boundaries of PSS7, PSS2, parasequence 17 (PS17) and PS14; the pore-cavity type of reservoir is constrained by the top boundaries of PSS7, PSS4, PS18 and PS12; and the pore type of reservoir is constrained by the top boundaries of PSS7, PSS6, PSS3, PSS2, PS18, PS17, PS14, and PS12. Seismic facies associated with shoals and mound-flat complexes are related to the facies distributions of pore cavities and pore reservoirs. MA1, MA3, MA7, and MA8 are predominant microfacies associations of favourable reservoirs of the Deng IV Member in the Gaoshiti-Moxi area. The above results are significant for further petroleum exploration and exploitation of ultradeep microbial carbonate reservoirs in this area.

## Introduction

In recent decades, microbial carbonate petroleum reservoirs have aroused worldwide interest, as numerous oil and gas fields have been discovered worldwide. Researchers have focused on not only the nature of the depositional and diagenetic characteristics of microbialites and associated facies but also the sedimentary and petrophysical properties of microbial carbonate reservoirs to better understand the origin, development, distribution, and depositional characteristics of such unique reservoirs^1^. Although a vast literature on microbes and their function in geological processes and products is available, only a small amount of literature addresses microbial carbonates and petroleum reservoirs, with few studies synthesizing the analysis of depositional characteristics with the results of favourable reservoir characteristics. Formed in a wide range of depositional settings, from nonmarine settings to marine environments of shallow to deep waters in Precambrian to Holocene time as the water conditions ranged from high energy to low energy, microbial carbonate reservoirs yield excellent potential for hydrocarbon productivity and have complex reservoir space systems and distributions.

**Figure 1:**
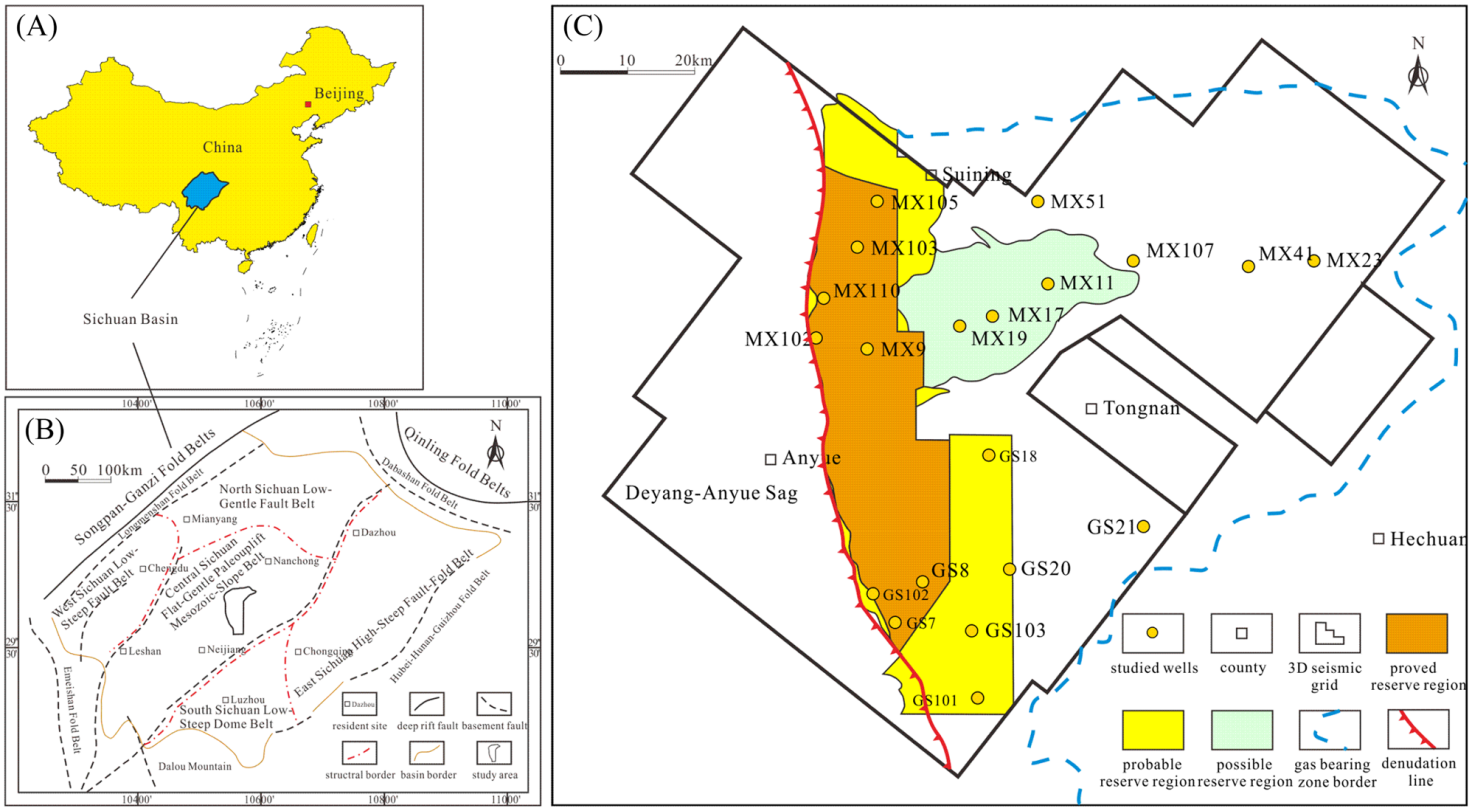
Location of the Gaoshiti-Moxi area (modified after Wang et al., 2021). (**a**) Location of the Sichuan Basin in China. (**b**) Structural division in the Sichuan Basin. (**c**) Petroleum exploration outline in the Gaoshiti-Moxi area.

In China, a large amount of oil and gas has been discovered in mainly Meso-Neoproterozoic microbial carbonate reservoirs, indicating brilliant prospects for Precambrian petroleum systems^2–5^. In particular, the Sinian Dengying Formation in the Sichuan Basin, formed in the late Ediacaran, has become a key microbial carbonate reservoir, with up to 10,000 × 10^8^ m^3^ of gas reserves^6^. The Sichuan Basin, in southwestern China, covers a large area of more than 19.2 × 10^4^ km^2^ (Fig. 1a). It is divided into five tectonic units, namely, the Central Sichuan Flat-Gentle Palaeouplift Mesozoic-Slope Belt, North Sichuan Low-Gentle Fault Belt, West Sichuan Low-Steep Fault Belt, East Sichuan High-Steep Fault-Fold Belt and South Sichuan Low-Steep Dome Belt (Fig. 1b)^7–9^. Microbial carbonate reservoirs are mainly distributed in the II Member and the IV Member of the Sinian Dengying Formation, which is subdivided into four members in the central Sichuan Basin, as well as the Middle Triassic Leikoupo Formation in the western Sichuan Basin, with thrombolites, prehnites, laminites and stromatolites as the main hydrocarbon reservoir rock types^2^. With multiple reservoir space types, including karst pores, cavities, and fractures, these microbial carbonate reservoirs generally have extremely high reservoir heterogeneity resulting from a long geological history, multistage tectonic movements and complicated diagenesis^10,11^. Many research papers have discussed the origin of reservoir heterogeneity from the microscale, such as pore structure characteristics, to the macroscale, such as paleogeomorphology^8,12^. However, the heterogeneity of microbial carbonate reservoirs is also closely related to their original sedimentary environments. Therefore, it is very important to systematically and comprehensively study the sedimentary influence on microbial carbonate reservoirs, from sequence architecture reconstruction to sedimentary facies analysis.

The Gaoshiti-Moxi area is the core area of the Anyue gas field, where the cumulative gas production reached over 81 billion cubic metres by December 2021 (Fig. 1c). The Deng IV Member, deposited on a carbonate ramp, is regarded as its crucial objective layer with a proven reserve of more than 680 billion cubic metres, accounting for 58% of the cumulative proven reserve by the end of 2021. The rock types of the Deng IV Member include light to dark grey algal dolomite, stromatolite, thrombolite, laminate, dolostone with intraclastic grains, crystalline dolostone, siliceous dolostone, lime–dolostone, brecciated dolostone, and dolomitic mudstone, among which the former four are associated with algae and are favourable for reservoirs^13,14^. In the reservoirs of the Deng IV Member, the reservoir space is composed of fenestral pores, interparticle dissolution pores, intercrystal dissolution pores, karst pores, cavities, and fractures^11^. Previous research results reveal that reservoirs of the Deng IV Member have superlow to low porosity and low permeability, with the average porosity of a single well between 2.21 and 3.95% and the average permeability of a single well between 0.0005 × 10^–3^ and 6.320 × 10^–3^ μm^2^ ^6,15^. At present, microbial mats, dolomitization and karstification are considered controlling factors on the formation of favourable reservoirs in the Deng IV Member. The microbial structures formed in widely distributed microbial mats are the origin of primary differences in reservoir qualities, whereas supergene karstification and the genesis of the Deyang-Anyue rift trough increased the reservoir heterogeneity^2^. With the deepening of the exploration and exploitation in the Gaoshiti-Moxi area, it remains a challenge to decipher the relationship between favourable reservoirs and the associated influences on their formation. Hence, it is very important to further our understanding of microbial carbonate reservoir characteristics and their relevance to depositional sequences and associated facies.

## Geological setting and stratigraphy

**Figure 2:**
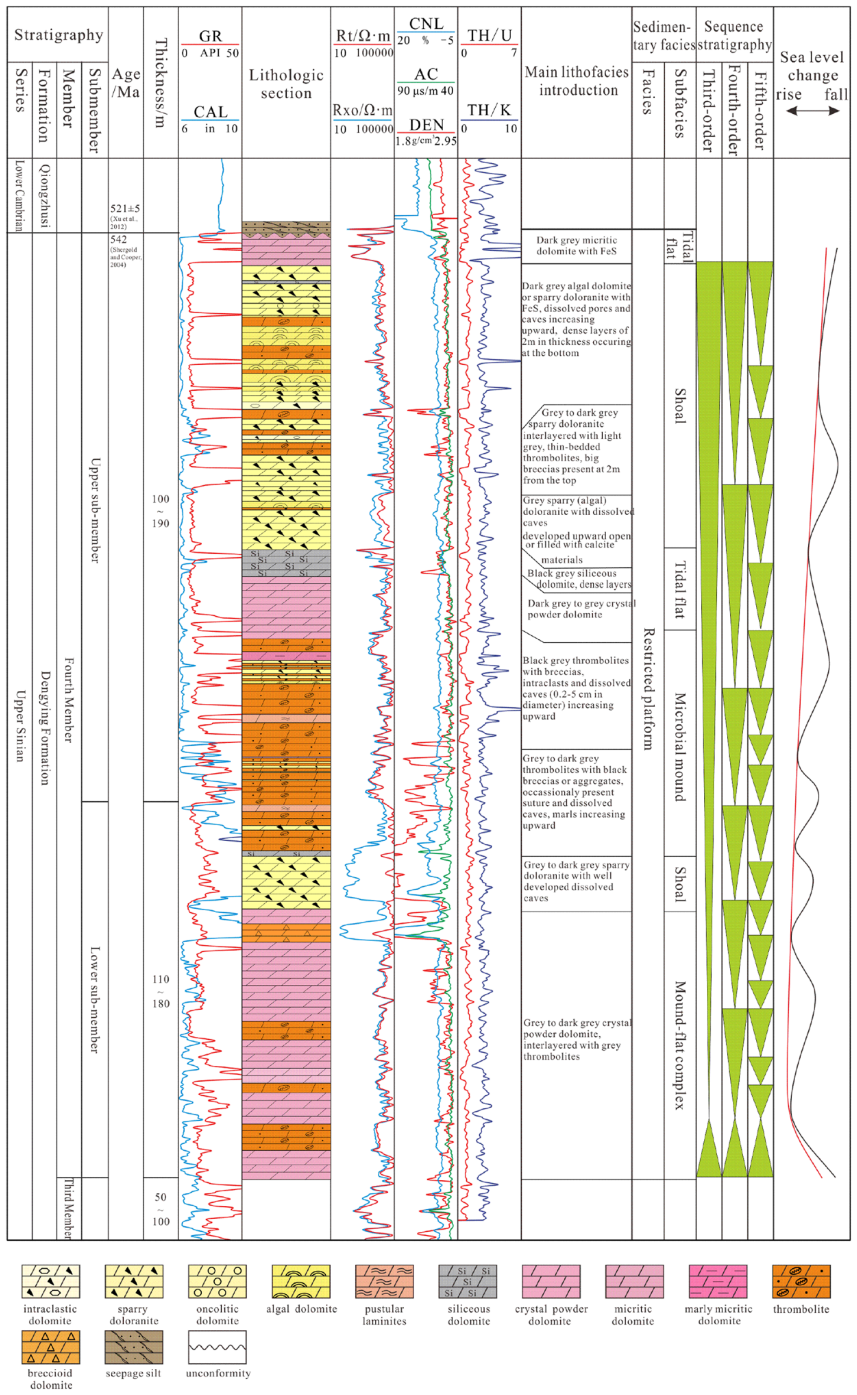
Sequence stratigraphic characteristics of the Deng IV Member in the Gaoshiti-Moxi area (modified after Wang et al. 2021).

The Deng IV Member deposited in the late Ediacaran, indicating the end of the global glacial period; this member shows evidence of one cycle of transgression and regression and was affected by episodes II and III of Tongwan movement, resulting in denudation to a large extent in the study area^16,17^. Hence, the overall thickness of the Deng IV Member ranges from 220 to 403 m, and it is thick in the northwest, thin in the southeast and disappears westwards from the Deyang-Anyue sag^18–24^. It unconformably contacts both the underlying (Deng III Member) and overlying strata (Maidiping Formation or Qiongzhusi Formation)^14^ (Fig. 2). Previous researchers have different views towards sequence division of the Deng IV Member according to well logging data: some believe that it comprises a highstand system tract of a 3rd order sequence and another complete 3rd order sequence^25^; some consider that it is a 3rd order sequence composed of transgressive systems tract (TST) and highstand systems tract (HST) as a whole^14,26^. Furthermore, seven parasequence sets (PSS1 to PSS7) within the 3rd order sequence have been identified with specific lithological cycles, bounded by instantaneously exposed punctuated surfaces or lithology and lithofacies transition surfaces^8^.

During the depositional period of the Deng IV Member in the study area, the climate was arid and hot, and a restricted carbonate platform developed under aragonite sea conditions^27–29^. In such an icehouse palaeoenvironment, light to dark grey microbial dolostone, dolostone with intraclastic grains, crystalline dolostone and other dolostones with different components or formation mechanisms, such as siliceous dolostone, lime–dolostone and brecciated dolostone, developed in the Deng IV Member. Featured by coccoid cyanobacteria and filamentous cryanobacteria, microbial dolostone is dominated by stromatolites, thrombolites and laminites taking on the form of microbial layers and mounds widely developed in intertidal and subtidal environments^30^. The dominant reservoir rock types are algae-cemented dolomite and algae-bounded dolarenite^8,31^. In terms of facies, six types of seismic facies and 16 microfacies were identified within the Deng IV member, representing shallow-water environments where microorganisms prefer to thrive, such as microbial mounds, shoals, build-up complexes, tidal flats (subtidal flats, subtidal-intertidal flats, intertidal-supratidal flats), and intermounds/shoals^14,32,33^. The facies change from marginal mound-beach complexes and inner shoal to tidal flat facies eastwards. In particular, on the platform margin, thick-bedded algal dolarenite, algae-cemented dolomite and algal stromatolitic dolomite interpreted as mound-bank complexes constitute the famous weathered Sinian karst reservoirs with a total thickness of 60–120 m^34,35^. Recent exploration in the study area has also suggested brilliant prospects in the interior platform, although this area is characterized by inner ramp deposits such as algal dolarenite, algal stromatolitic dolomite, and crystalline dolostone interlayered with siliceous dolostone^12,34^.

## Material and methods

The data used in this study are 3D seismic data that cover the whole study area, well logs (conventional logs, natural gamma spectroscopy logs, and formation microresistivity image (FMI) logs) from 15 wells, 1300 m of cores from 20 wells, and nearly 1000 thin sections. Moreover, reservoir layer data from 46 wells and reservoir physical property data from 20 wells are also utilized in the reservoir analysis. For different types of reservoirs, a thickness-weighted calculation is applied by the sum product of net pay thickness and porosity or permeability dividing the sum of net pay thickness in each parasequence set. The integrated interpretation of 3D seismic profiles in the study area is employed to reveal the seismic facies distribution and document the geomorphology of depositional facies after being calibrated with well data. A seismic root-mean-square (RMS) amplitude slice is made with a 60 μs time window below the top surface of the Deng IV Member and is overlapped by distribution maps of effective reservoirs and a seismic facies map of the Deng IV Member for potentially favourable reservoir prediction. Well logging data are used for the division of high-frequency sequences and system tracts, as well as reservoir strata correlation. High-resolution FMI interpretations, as well as colour, sedimentary structure, depositional texture, grain type and size, and fossil (algae) core and thin section data, are used mainly as proxies for the identification of reservoir microfacies and microfacies associations and reservoir spaces, such as pores, karst caves and fractures. In particular, the reservoir layer data are applied in the physical property analysis of each reservoir type in the sequence framework and microfacies associations, as well as in defining the reservoir distribution boundary. In addition, the porosity and permeability values are obtained by laboratory measurements from rock samples of different types of reservoirs, reflecting different microscopic structures of interconnected pore spaces.

## Reservoir characteristics

### Reservoir types and physical properties

Regionally, laminite and stromatolitic dolomites are the most important in the Deng IV Member, with three reservoir intervals that are 119 m thick in total^36^. In the study area, reservoirs are developed in the upper submember of the Deng IV Member and dominated by microbial carbonate deposits from carbonate ramps, such as boundstone associated with thrombolitic buildups, algae-cemented dolomite, algal stromatolitic dolomite and algal dolarenite^37–40^. According to the reservoir space characteristics, effective reservoirs in the Deng IV Member featuring weathering crust karst are mainly classified into three types, including fracture-cavity reservoirs, pore-cavity reservoirs and pore reservoirs, with cut-off porosities and permeabilities of 2.6% and 0.13 × 10^–3^ μm^2^, respectively^41,42^. The reservoir space is genetically mostly secondary, with the most common types of reservoir spaces being residual intragranular pores, intergranular pores, intercrystalline pores and cavities.

**Figure 3:**
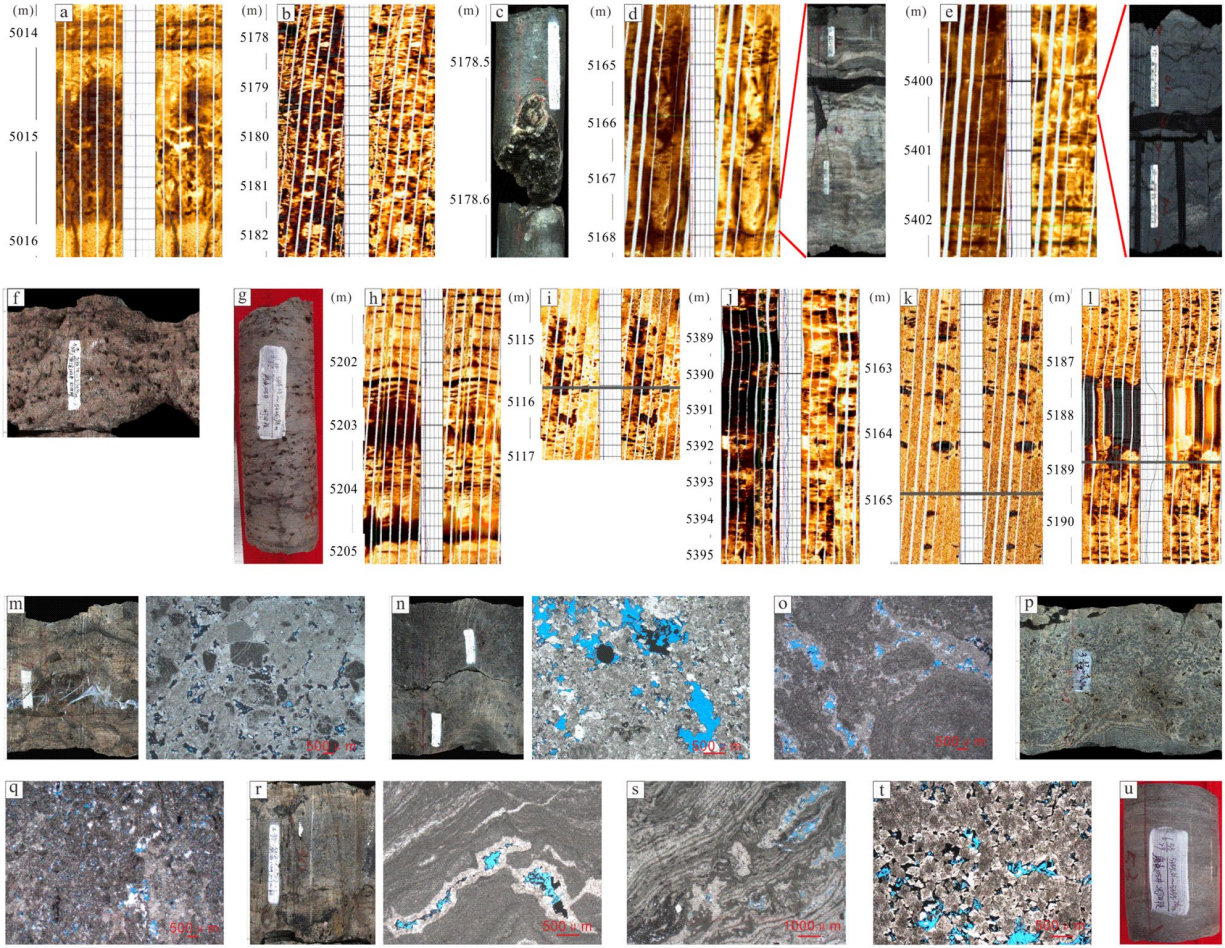
FMI and core characteristics of reservoirs in the Gaoshiti-Moxi area. (**a**) Fracture-cellular cavity, dolarenite, well MX9, 5313.75–5316.20 m. (**b**) Fracture-parallel pores, thrombolite, well MX19, 5177.37–5482.42 m. (**c**) Fracture-parallel cavity, micritic crystalline dolomite, well MX41, 5163.98–5168.40 m. (**d**) Fracture-parallel cavity, algal dolarenite, well MX51, 5222.8–5228.6 m (FMI image), 5367.39–5367.87 m (core). (**e**) Fracture-layered cavity, marly micritic dolomite, well MX51, 5398.90–5402.50 m (FMI image), 5400.31–5400.53 m (core). (**f**) Stromatolite-parallel pores and cavities, stromatolite, well MX102, 5194.78–5194.90 m. (**g**) Laminae-parallel pores, laminite, well MX105, 5325.68–5325.98 m. (**h**) Laminae-parallel pores and cavities, micritic dolomite, well GS18, 5163.98–5168.40 m. (**i**) Cellular-shaped pores and cavities, thrombolite, well MX19, 5114.10–5117.05 m. (**j**) Random-shaped pores and cavities, micritic crystalline dolomite, well GS21, 5387.62–5395.25 m. (**k**) Toruloid-shaped pores and cavities, thrombolite, well GS7, 5161.78–5166.00 m. (**l**) Pendulous-shaped pores and cavities, thrombolite, well GS20, 5185.60–5190.80 m. (**m**) intragranular pores, dolarenite, well GS102, 5096.47–5096-75 m. (**n**) Residual intragranular pores, thrombolite, well GS102, 5137.54–5137.83 m. (**o**) Residual intergranular pores, algal dolarenite, well MX108, 5295.97 m. (**p**) Intergranular pores, algal dolarenite, well GS111, 5322.35–5322.65 m. (**q**) Dissolved pores in thrombolite, well MX108, 5297.53 m. (**r**) Dissolved pores in the shape of lamina, laminite, well MX108, 5317.06–5317.22 m. (**s**) Dissolved pores in stromatolite, well MX108, 5306.79 m. (**t**) Intercrystalline pores, silty-fine crystalline dolomite, well MX 109, 5126.31 m. (**u**) Pinhole pores, laminite, well MX105, 5355.61–5355.76 m.

**Figure 4:**
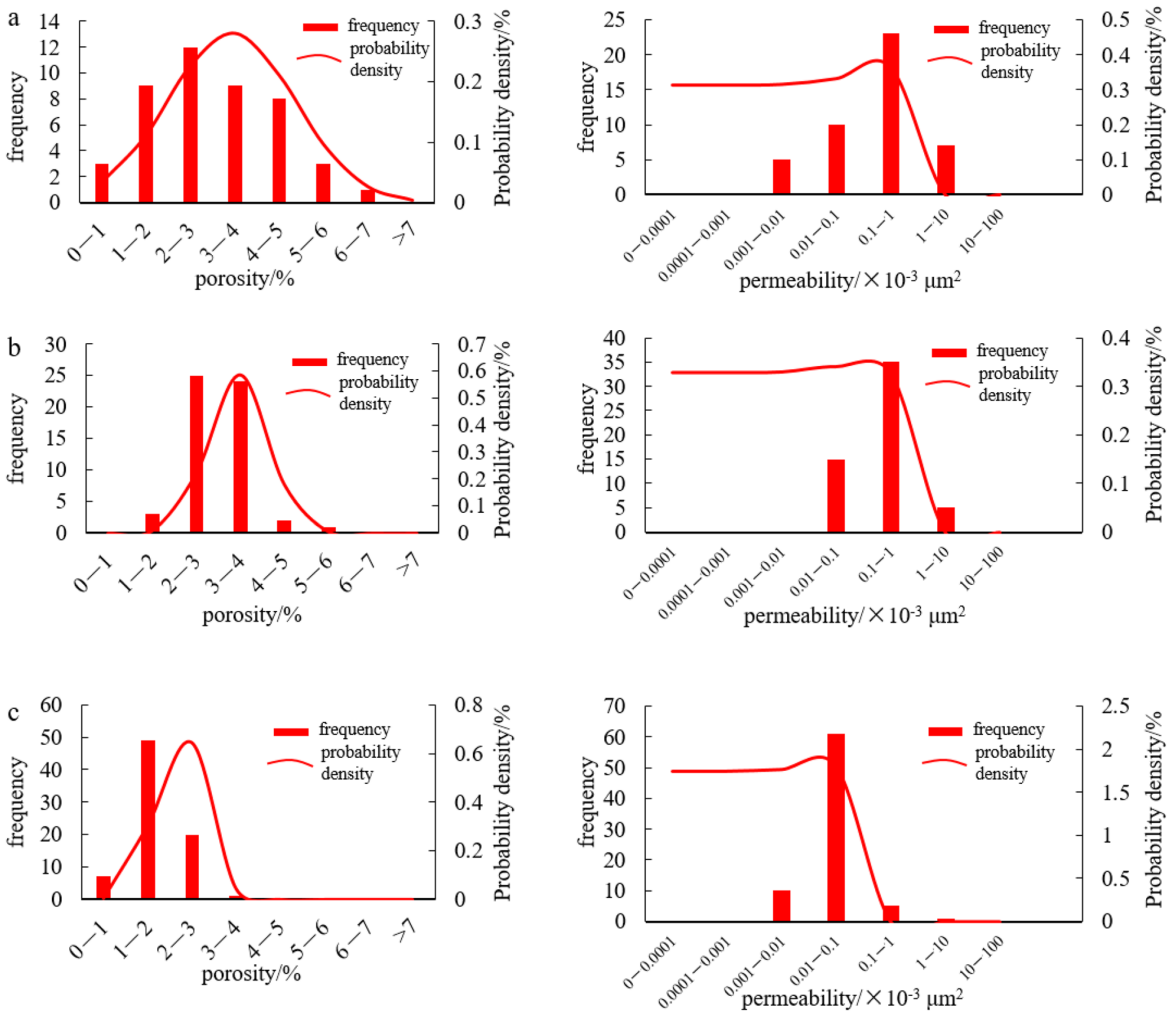
Porosity and permeability frequency histograms and probability density plots of reservoirs in the Gaoshiti-Moxi area: (**a**) Porosity and permeability frequency histogram and probability density plot of the fracture-cavity reservoir type. (**b**) Porosity and permeability frequency histogram and probability density plot of the pore-cavity reservoir type. (**c**) Porosity and permeability frequency histogram and probability density plot of the pore reservoir type.

Here, a fracture-cavity reservoir is dominated by thrombolite dolomite and algae-bounded dolarenite, followed closely by marly micritic dolomite and microcrystalline dolomite (Fig. 3a–e). In the FMI image, it is obvious that cavities and fractures are well matched. In addition, this type of reservoir can be further divided into fracture-cellular cavity reservoirs (Fig. 3a), fracture-parallel fracture-cavity reservoirs (Fig. 3b–d), and fracture-layered cavity reservoirs (Fig. 3e) by the distribution pattern and shape of the fractures and cavities. The porosity of a fracture-cavity reservoir is mainly distributed from 1 to 5% (Fig. 4a), and the permeability is 0.01–1 × 10^–3^ μm^2^ (Fig. 4a).

A pore-cavity reservoir features microbial dolomite in which fractures are less developed, such as thrombolite dolomite, stromatolitic dolomite and laminite dolomite (Fig. 3f–l). The stromatolite-parallel and lamina-parallel pore-cavity reservoirs are categorized into parallel-layered pore-cavity reservoirs (Fig. 3f–h). In addition, cellular, random, toruloid, pendulous shaped cavities are widely developed. In local areas, dark laminae are dissolved to form fabric-selective pores (Fig. 3i–l). The porosity of a pore-cavity reservoir is mainly distributed from 2–4% (Fig. 4b), and the permeability is 0.01–1 × 10^–3^ μm^2^ (Fig. 4b).

The pore reservoir is characterized by dolarenite, algal dolarenite and thrombolite dolomite followed by micritic-fine crystalline dolomite, laminite dolomite and stromatolitic dolomite (Fig. 3m–u). In cores composed of residual intragranular pores (Fig. 3m,n), intergranular pores (Fig. 3o–r), and intercrystalline pores in the shape of laminae or pinholes (Fig. 3s–u), dissolved pores are more prevalent than fractures and cavities. Thin sections show that tube-shaped coccoid cyanobacteria, stromatolite-related microorganisms, dendritic, and hammer-ball and filamentous cyanobacteria are widespread (Peng et al. 2014). The porosity of the pore reservoir is mainly distributed between 1 and 3% (Fig. 4c), and the permeability is distributed between 0.001 × 10^–3^ and 0.1 × 10^–3^ μm^2^ (Fig. 4c).

**Figure 5:**
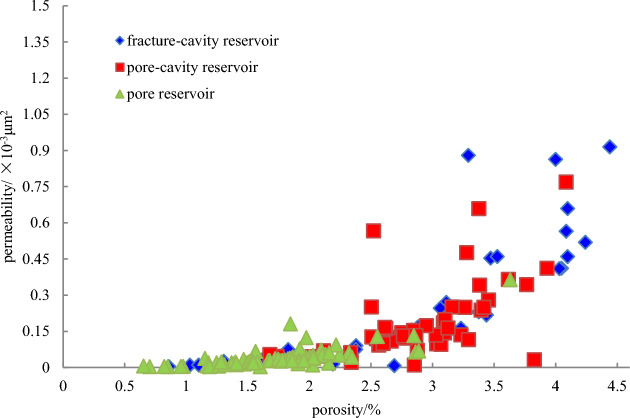
Relationship between reservoir porosity and permeability in the Deng IV Member in the Gaoshiti-Moxi area.

On the whole, the studied reservoirs mainly have low porosity and low permeability with strong heterogeneity. The physical properties of fracture-cavity and pore-cavity reservoirs are generally better than those of pore reservoirs. According to statistical data from 177 full-diameter cores of reservoirs in the Deng IV Member from 20 wells, the porosities of fracture-cavity and pore-cavity reservoirs are all above 2% and below 3% of the pore reservoir; the permeabilities of fracture-cavity and pore-cavity reservoirs are above 0.1 × 10^–3^ μm^2^ and below 0.15 × 10^–3^ μm^2^ of the pore reservoir. Moreover, the statistical data indicate that the permeability of the fracture-cavity exceeds that of the pore-cavity when the porosity is above 3.2% (Fig. 5).

**Figure 6:**
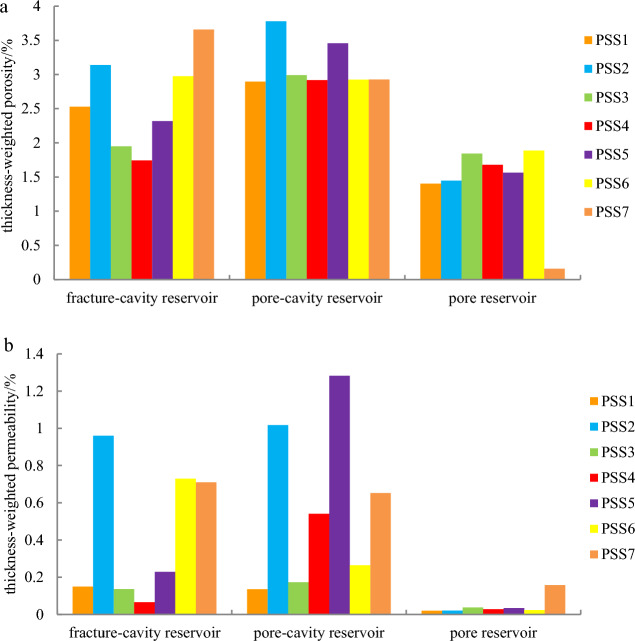
Thickness-weighted reservoir porosity and permeability histograms of PSS1- PSS7. (**a**) Thickness-weighted reservoir porosity histogram of PSS1-PSS7. (**b**) Thickness-weighted reservoir permeability histogram of PSS1-PSS7.

The physical properties of the three types of reservoirs are also compared vertically from parasequence set 1 (PSS1) to parasequence set 7 (PSS7) based on the previous sequence division scheme of Wang et al. 2021 (Fig. 6). The physical properties of the fracture-cavity reservoir are better in both the upper and lower parts of the parasequence sets (PSS1-PSS2, PSS6-PSS7) and worse in the middle part (PSS3-PSS5) (Fig. 6). Regarding the pore-cavity reservoir, its porosity is high in general, above 2.8% in all parasequence sets (Fig. 6a), and permeability is above the cut-off value of 0.13 × 10^–3^ μm^2^ (Fig. 6b). For the pore reservoir, its porosity is below 2% in each parasequence set (Fig. 6a), and the permeability is below 0.1 × 10^–3^ μm^2^ in the parasequence sets despite PSS7 (Fig. 6b).

### Reservoir distribution

**Figure 7:**
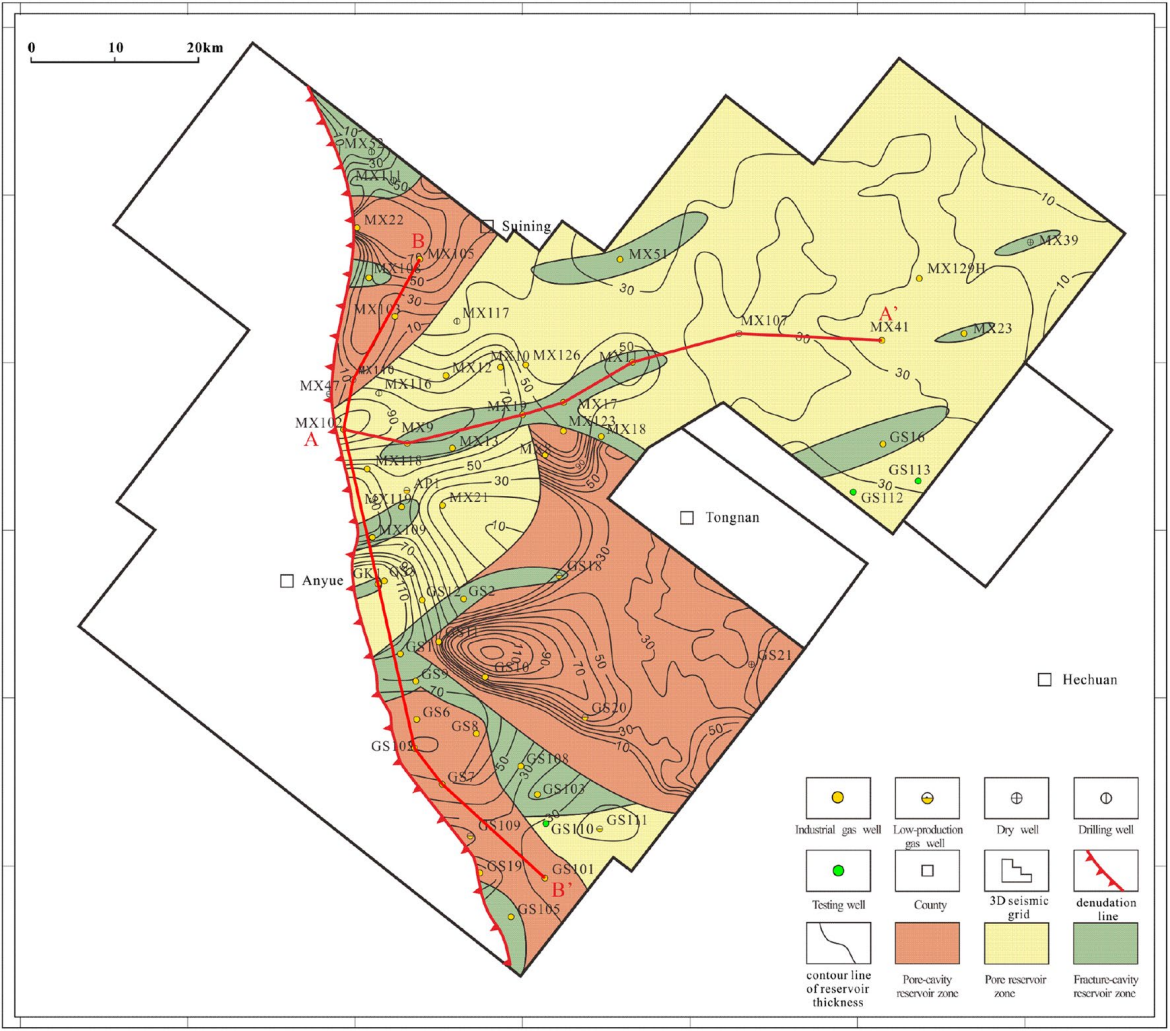
Reservoir distribution in the Deng IV Member in the Gaoshiti-Moxi area.

**Figure 8:**
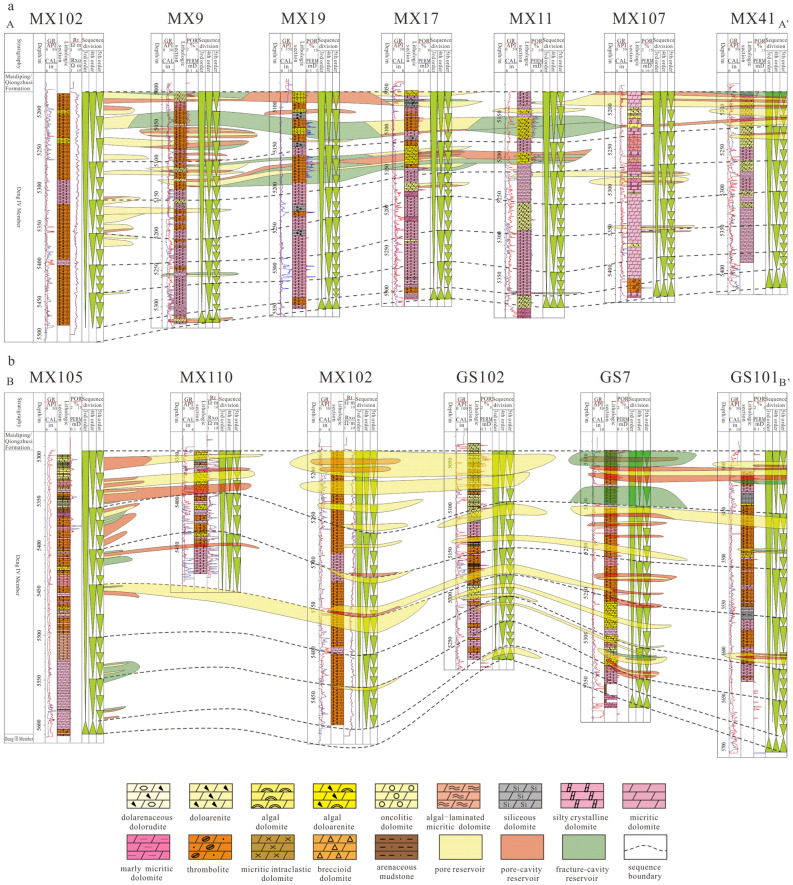
Reservoir correlation in the Deng IV Member in the Gaoshiti-Moxi area: (**a**) Reservoir correlation in well MX102-MX9-MX19-MX17-MX11-MX107-MX41. (**b**) Reservoir correlation in well MX105-MX110-MX102-GS102-GS7-GS101.

Vertically, the above three types of reservoirs are mainly developed in PSS5-PSS7 according to previous research results on sequence division^14^. Laterally, fracture-cavity reservoirs are distributed sporadically in the shape of short stripes in the study area. Generally, at isolated wells MX53, MX51, MX39, MX23, MX119, GS16 and GS105, only small-scale fracture-cavity reservoirs are developed (Fig. 7). However, in well zone MX9-MX13-MX19-MX17-MX18-MX11, a large-scale distributed fracture-cavity reservoir is continuously developed (Fig. 8a). Fracture-cavity reservoirs are also developed in PSS2-PSS3 in s local region of the Gaoshiti area, including at wells GS7, GS8, GS20, and GS21 (Fig. 8b).

A pore-cavity reservoir occupies large areas in Gaoshiti and northern Moxi and is stable in distribution (Fig. 7). Vertically, it is superimposed, and its continuity is better in the upwards sequences. In particular, it is successively developed in GS7 and GS20. In addition, in PSS4 of wells MX107, GS103, and GS101 and PSS1-PSS3 of wells MX105, MX9, GS7, GS103, and GS20, thinner and less continuous pore-cavity reservoirs are developed (Fig. 8).

A pore reservoir is mainly distributed in the Moxi area with an increasing trend of thickness upwards (Fig. 7). In contrast, in the Gaoshiti area, pore reservoirs are only developed in wells GS110 and GS111. In addition, in PSS1-PSS4 of well zones GS102-GS7-GS101, GS102-GS8-GS103, and MX105-MX102, thin and isolated pore reservoirs are developed (Fig. 8b).

## Depositional influence on the reservoir

### Depositional sequence effects on the reservoir

**Figure 9:**
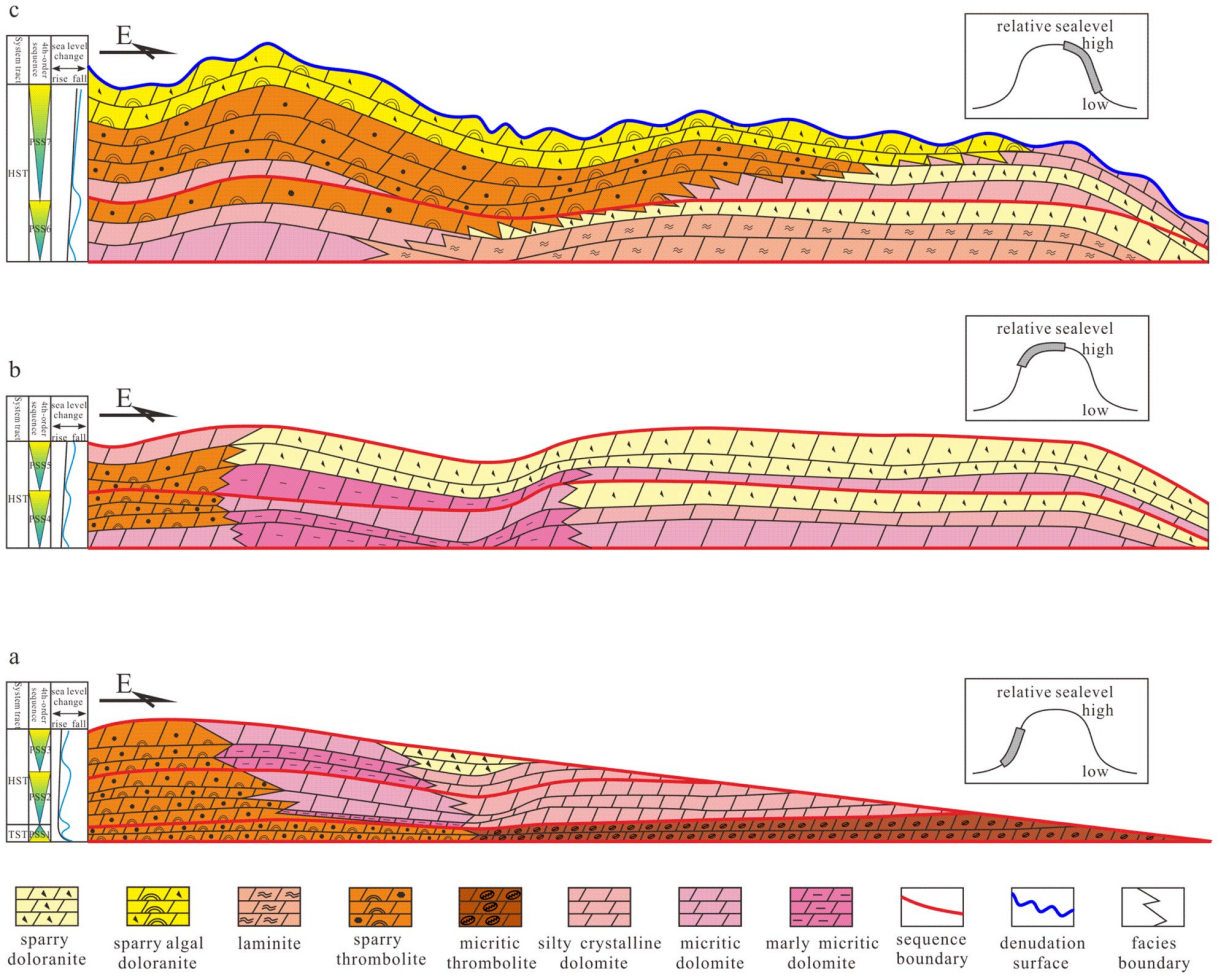
Development model of the high-frequency sequence in the Deng IV Member in the Gaoshiti-Moxi area: (**a**) Stacking pattern of PSS1-PSS3. (**b**) Stacking pattern of PSS4-PSS5. (**c**) Stacking pattern of PSS6-PSS7.

Previous studies have shown that the Deng IV Member can be divided into 7 parasequence sets (PSS1-PSS7)^14^. During the depositional period of PSS1-PSS3, high-frequency sequences were stacked in a retrogradation type, with the sea level changing from falling rapidly to rising. Influenced by inherited palaeogeomorphology, sparry thrombolites, microcrystalline dolomites and micritic dolomites are well developed on the platform margin; micritic thrombolites and micritic dolomites are well developed on the inner platform (Fig. 9a). During the depositional period of PSS4-PSS5, high-frequency sequences were stacked in an aggradation type as the sea level rose rapidly. On the platform margin, sparry thrombolites, micritic dolomites or argillaceous micritic dolomites were well developed; in the platform interior, micritic dolomites followed by sparry intraclastic dolomites were well developed (Fig. 9b). During the depositional period of PSS6-PSS7, stacked in a progradation type as the sea level changed from rising slowly to falling slowly, high-frequency sequences were composed of sparry thrombolites, intraclastic dolomites, algal dolomite, and microcrystalline dolomites on the platform margin and microcrystalline dolomites, micritic dolomites, sparry intraclastic dolomites and laminites in the platform interior, bearing favourable reservoir layers (Fig. 9c). In the late Ediacaran, affected by episode II of the Tongwan movement, the Deng IV Member was subjected to various degrees of weathering and denudation.

**Figure 10:**
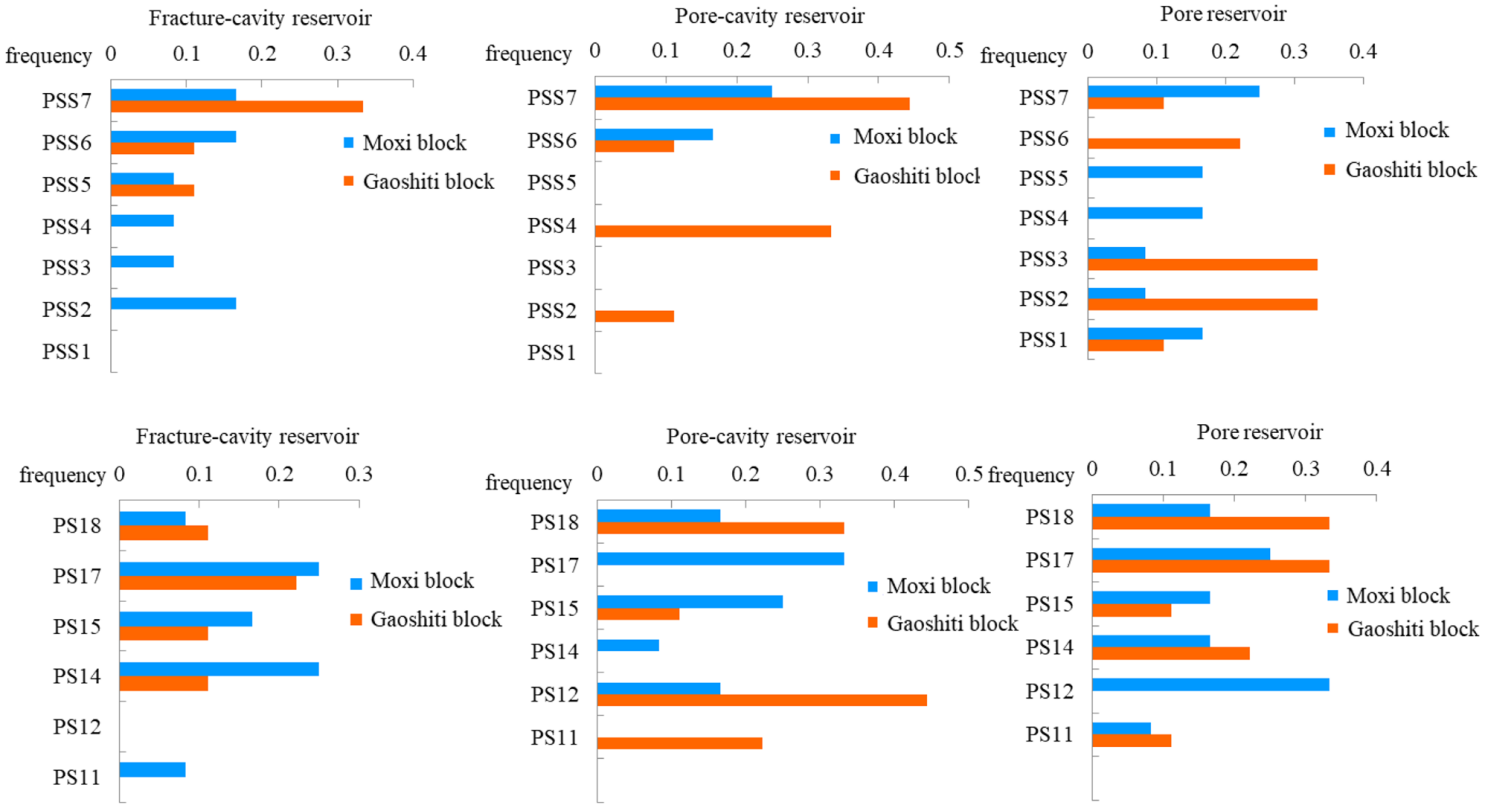
Frequency bar graphs of wells where reservoirs developed around the boundary of each parasequence and parasequence set in the Deng IV Member in the Gaoshiti-Moxi area.

According to the statistical results of the reservoir types developed around the boundary of each parasequence set from 21 wells in the study area, the fracture-cavity type of reservoir is constrained by the top boundaries of PSS7, PSS2, PS17 and PS14, with a main porosity between 1 and 5% and permeability of 0.01–1 × 10^–3^ μm^2^ (Figs. 4a, 10); the pore-cavity type of reservoir is constrained by the top boundaries of PSS7, PSS4, PS18 and PS12, with a main porosity between 2 and 4% and permeability of 0.01–1 × 10^–3^ μm^2^ (Figs. 4b, 10); and the pore type of reservoir is constrained by the top boundaries of PSS7, PSS6, PSS3, PSS2, PS18, PS17, PS14, and PS12, with a main porosity between 1 and 3% and permeability between 0.001 × 10^–3^ and 0.1 × 10^–3^ μm^2^ (Figs. 4c, 10).

### Effects of seismic facies on reservoirs

**Figure 11:**
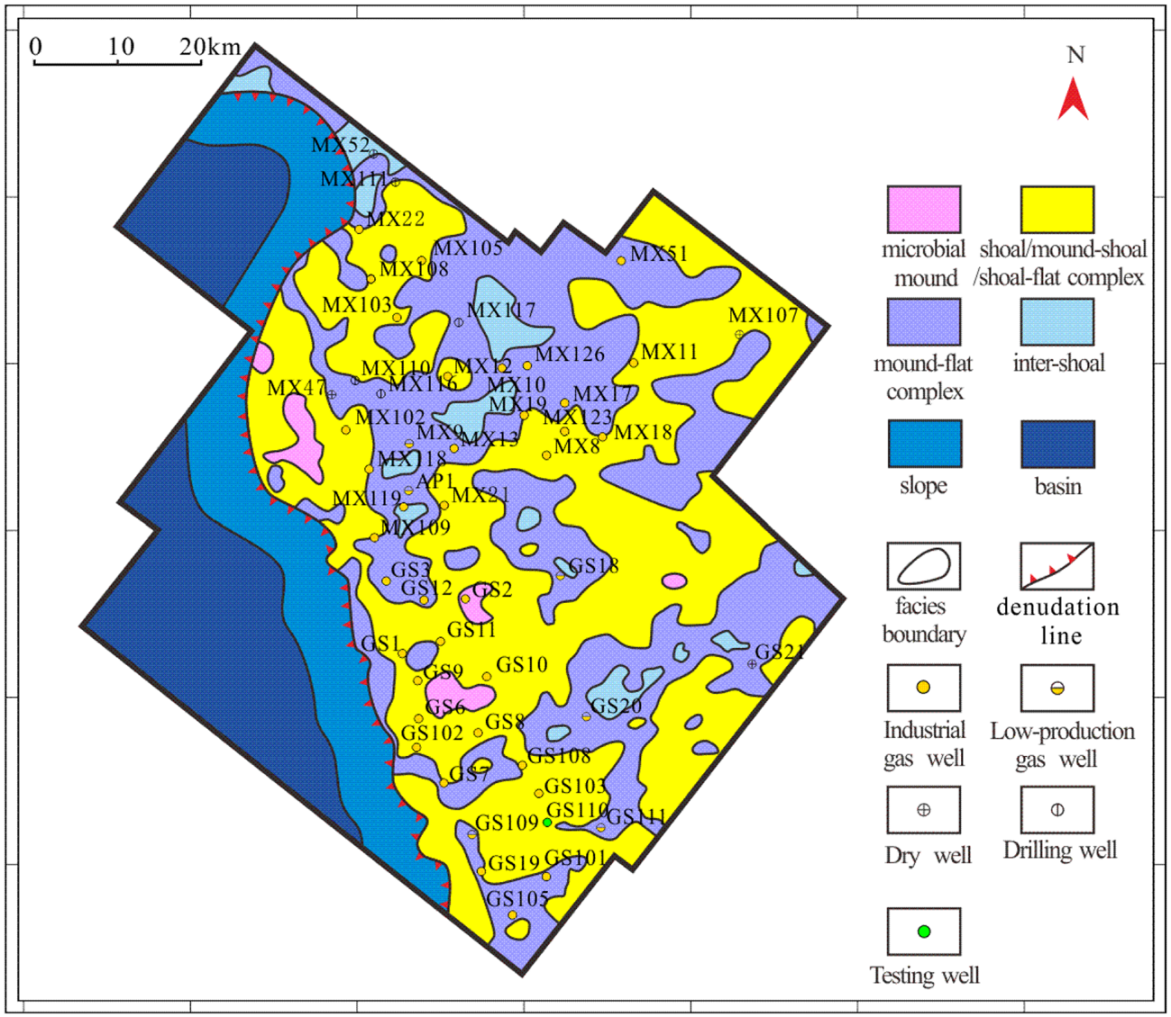
Seismic facies map of the Deng IV Member in the Gaoshiti-Moxi area.

Six seismic facies have been recognized in the study area, representing microbial mound, shoal, mound-shoal complex, mound-flat complex, shoal-flat complex, and intershoal/mound sedimentary facies^14^. They have been identified in well profiles with cross-sectional seismic facies analysis and sedimentary facies analysis^14^. Furthermore, it has been found that the average energy, average magnitude and mean amplitude are well correlated with certain sedimentary facies identified in the well, such as microbial mound facies, mound-flat complex facies, intershoal/mound facies, and facies association closely related to the shoal facies, so they are chosen as effective attributes to make a neural net map with a 30 ms time window below the top surface of the Deng IV Member, which constitutes the principal part of the map of seismic facies distribution (Fig. 11).

By comparing the map of the reservoir distribution and the map of the seismic facies distribution, we believed that the facies of most pore-cavity reservoirs belong to facies-associated shoals, such as shallow shoals, mound-shoal complexes, and shoal-flat complexes; the facies of most pore reservoirs belong to mound-flat complexes. Regarding fracture-cavity reservoirs, it seems that the seismic facies has little influence on their distribution.

### Microfacies association effects on reservoirs

**Figure 12:**
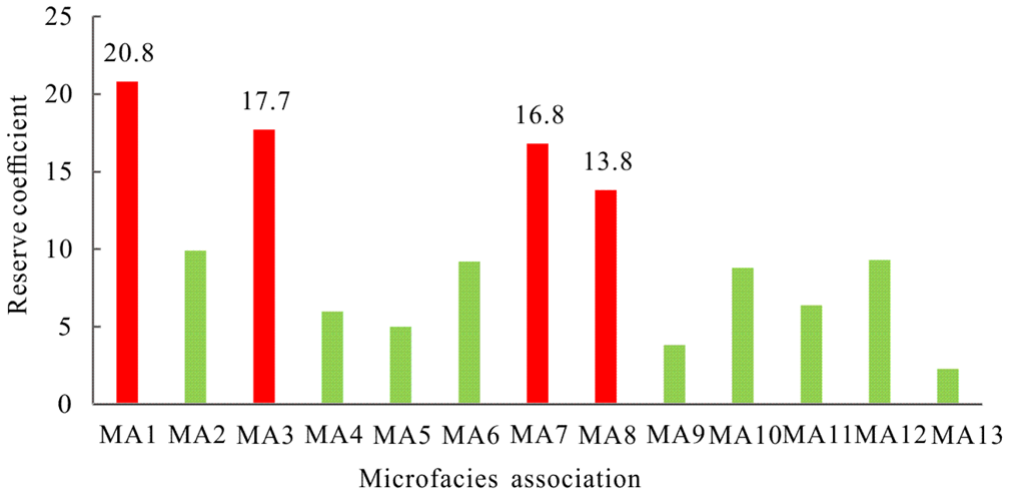
Reservoir coefficient histogram of microfacies associations in the Gaoshiti-Moxi area.

Thirteen types of microfacies associations (MA1-MA13) have been identified in the study area, representing distinctive depositional facies successions comprising subtidal microbial mound and shoal, tidal flat, intershoal/microbial mound, mid-ramp and meteoric freshwater deposits^14^. A reserve coefficient is a common parameter that reflects the degree of petroleum enrichment in a well in the early stage of reservoir production for reservoir block optimization and well production prediction. It is more suitable than the formation coefficient for describing low-permeability reservoirs^54-55^. After comparing reserve coefficient values expected for the thirteen types of microfacies associations in 9 wells, MA1, MA3, MA7, and MA8 are clearly predominant microfacies associations of favourable reservoirs of the Deng IV Member in the Gaoshiti-Moxi area with values of 20.8, 17.7, 16.8, and 13.8, respectively (Fig. 12).

**Figure 13:**
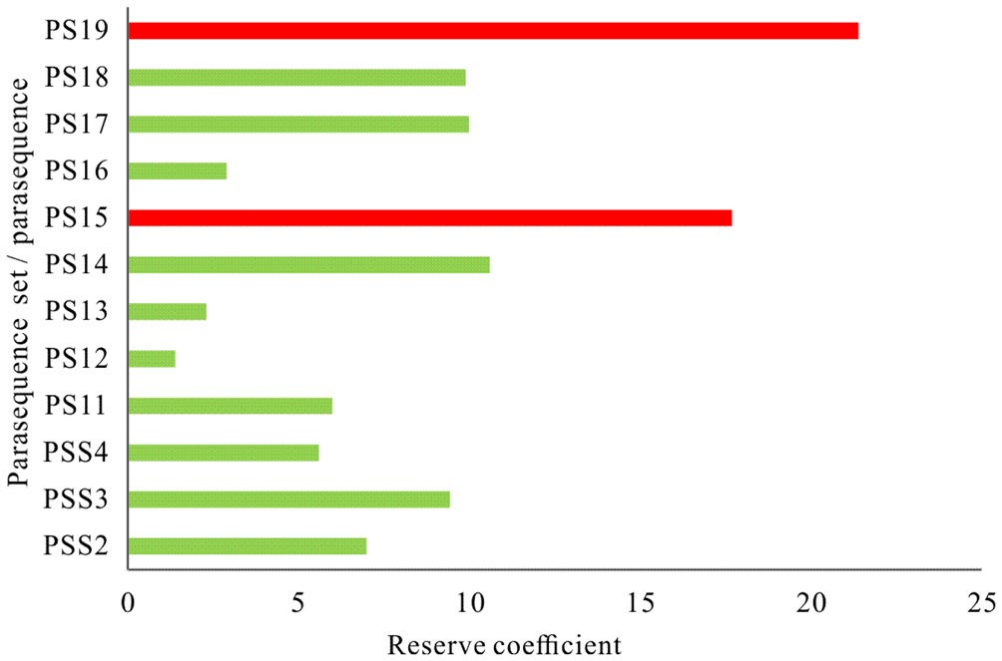
Reserve coefficient bar graphs of each parasequence and parasequence set in the Deng IV Member in the Gaoshiti-Moxi area.

**Figure 14:**
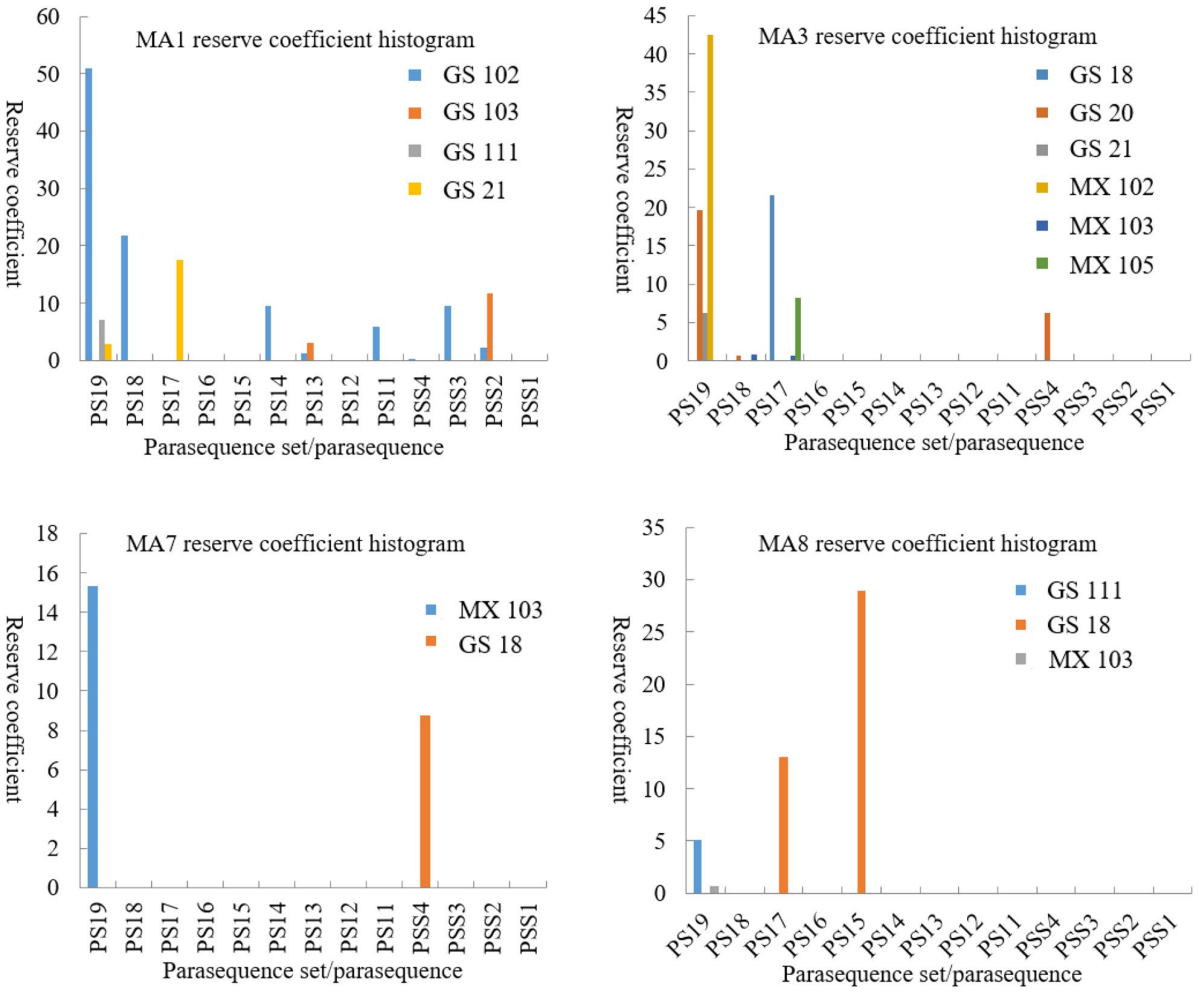
Reserve coefficient histograms of MA1, MA3, MA7, and MA8 in each parasequence and parasequence set in the Deng IV Member in the Gaoshiti-Moxi area.

On the other hand, favourable reservoirs are probably developed in PS19 and PS15 in terms of high values of reserve coefficient expectation in parasequences and parasequence sets in the 9 wells (Fig. 13). Moreover, the above values of reserve coefficient expectation in MA1, MA3, MA7, and MA8 are also statistical data of the parasequence and parasequence sets applied for comparison. Finally, it is proven that MA1, MA3, and MA7 have positive effects on creating favourable reservoirs in PS19, which corresponds to the top of PSS7; MA8 has a positive effect on creating favourable reservoirs in PS15, which corresponds to the top of PSS6 (Fig. 14). MA1, MA3, MA7 and MA8 are all arranged in a shalowing-upwards metre-scaled sequence^14^. Composed of grey to dark grey sparry thrombolites, typically capped by thick layers of grain dolomites, MA1 (Mf4-Mf1-Mf3) is mainly present in PSS2 ~ PSS7 in the Gaoshiti area with a thickness of approximately 15 m and appears to be common on the platform margin from PSS4^14^, representing an open high-energy shallow subtidal environment varying from subtidal microbial mound to shoal. Characterized by grey to dark grey sparry thrombolites and stromatolites with millimetre-scale undulate, domal and parallel laminations upward, assemblage MA3 (Mf4-Mf12-Mf11) is present in the platform interior of the Gaoshiti area, and only PSS7 is present on the platform margin of the Moxi area with a thickness of approximately 5 m, representing depositional settings from a subtidal microbial mound to an intertidal flat and then to a subtidal flat. With dark grey thick-bedded micritic dolarenites stacked by algal aggregate dolomites as most of the succession and grey marly dolomites and undulate stromatolites as the uppermost part, assemblage MA7 (Mf7-Mf6-Mf12-Mf9) is present in PSS4 ~ PSS6 in the platform interior of the Gaoshiti area and in PSS7 on the platform margin of the Moxi area with a thickness of approximately 5 m^14^, reflecting the change in depositional setting from low-energy subtidal shoal to intertidal flat. Featured by very thin layers of both sparry thrombolites and oncolitic dolomites and thick layers of microbial laminites and crystalline dolomites, assemblage MA8 (Mf4-Mf2-Mf10-Mf14) is present in PSS5 ~ PSS7 on the platform interior of the Gaoshiti area and in PSS6 ~ PSS7 on the platform margin of the Moxi area with a thickness of approximately 5 m^14^, reflecting a complex depositional setting variation from a high-energy subtidal microbial mound to shallow shoal, then to an intertidal flat and finally to a supratidal flat.

## Favourable reservoir prediction

**Figure 15:**
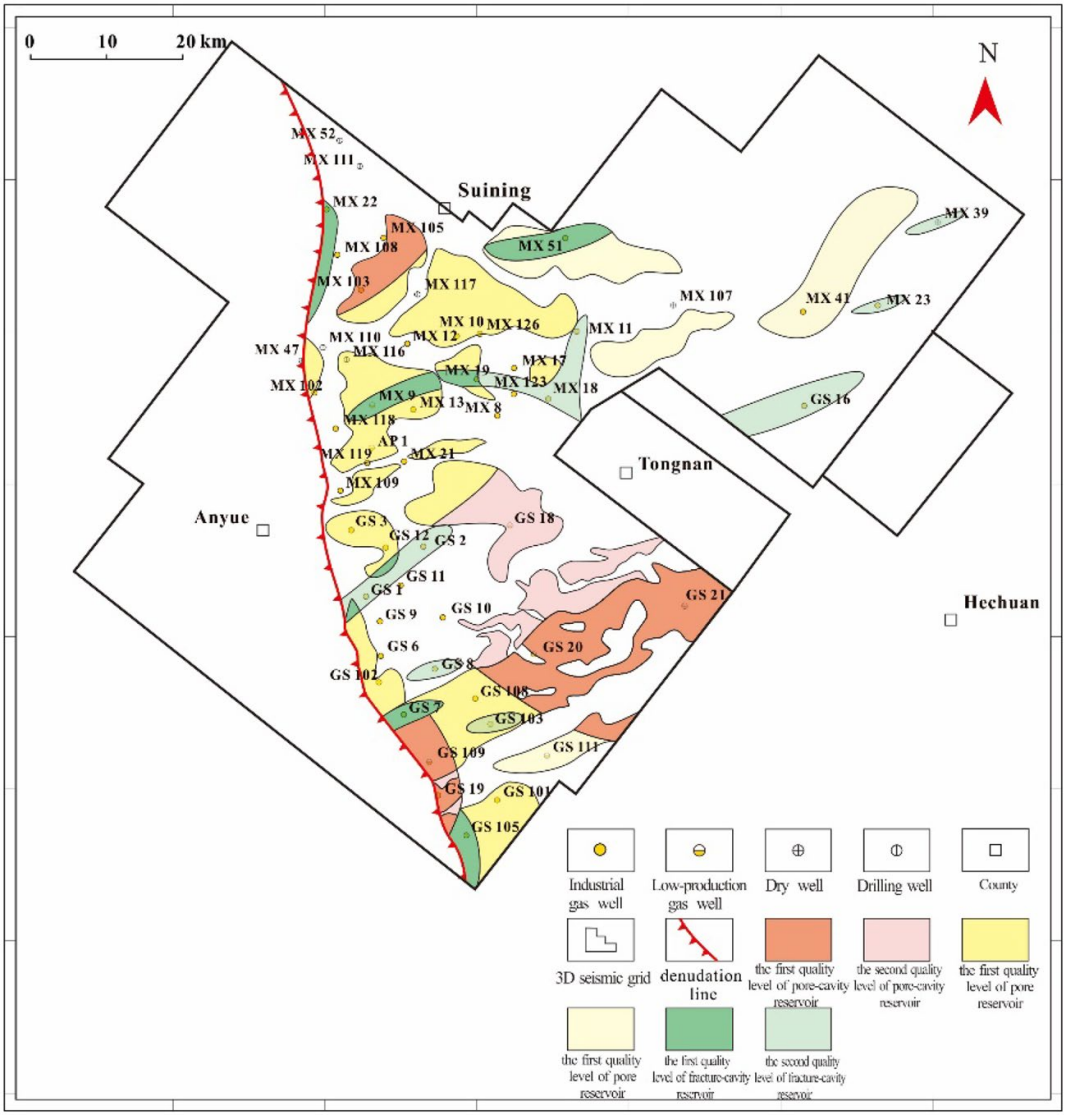
Map of favourable reservoir prediction in the Deng IV Member in the Gaoshiti-Moxi area.

The seismic RMS amplitude has a positive relationship with rock density, and this seismic attribute is often applied in lithology identification^43,44^. In the study area, two quality levels of the three types of reservoirs with nearly 40 potential blocks are determined according to the strong and weaker RMS amplitude areas within each type of reservoir distribution zone (Fig. 15). The potential fracture-cavity reservoirs are sporadically distributed on a relatively small scale. The first quality level of fracture-cavity reservoirs is distributed in the MX22, MX9, MX19, MX51, GS7, and GS105 well zones; the second quality level is distributed in the MX18, MX23, MX39, GS1-GS2, GS8, GS16, and GS103 well zones. The potential pore-cavity reservoirs are predominantly distributed in the Gaoshiti area. The first quality level of pore-cavity reservoirs is distributed in the MX103, GS20, and GS21 well zones; the second quality level is distributed in the GS18 well zone and GS20 well zone to the west in the shape of a northeast‒southwest oriented stripe. The potential pore reservoirs are relatively vastly distributed. The first quality level of pore reservoirs is distributed on the platform margin; the second quality level is distributed in GS111, around MX51, and in the southern MX107 well zones.

## Discussion

Reservoirs in the Deng IV Member exhibit extremely high heterogeneity and complexity since they are ultradeep carbonate reservoirs that have undergone a series of events during a long burial period, from sedimentation, diagenesis, and tectonism to hydrocarbon accumulation. On the other hand, the Anyue gas field has now entered a stage of maturing development, showing large heterogeneity in well productivity in the development process^45^. Hence, it remains a major challenge to clarify the complex reservoir genesis and distribution. In this paper, we focus on sedimentary effects on reservoir development and distribution for better reservoir prediction based on our previous study results of sequence division and characteristics and depositional facies identification and description in the Deng IV Member^14^. Due to the lack of production and dynamic monitoring data and data on the percolation characteristics and connectivity analysis, this new approach seems less satisfactory for obtaining a synthetic but reasonable reservoir prediction. Hence, our research methods of favourable reservoir prediction are more applicable for the early exploration of a new block to some extent.

Moreover, the palaeogeomorphology of the Deng IV Members in the Gaoshiti-Moxi area should be considered as another key factor for favourable reservoir prediction in previous studies43, 59, 25. The drilling results reveal that the formation of both fracture-cavity and pore-cavity reservoirs is closely related to karstic slope and karstic platform-slope break areas. Our earlier map of the seismic facies distribution has been overlapped by a map of the palaeogeomorphological distribution of the study area by Yan et al. This map shows that on the landform unit of the karst platform surface, a mound-flat complex is well developed in the Moxi area; facies associated with shoals, such as shallow shoals, mound-shoal complexes and shoal-flat complexes, are well developed in the Gaoshiti area, implying a gentle karst landform in the Moxi area and a steep karst landform in the Gaoshiti area. Meanwhile, in the two major palaeogrooves present on the palaeogeomorphological distribution map by Yan et al., the intershoal/mound is well developed in the Moxi area, and the mound-flat complex is well developed in the Gaoshiti area, corresponding to the obvious palaeogeomorphological differences between the Moxi and Gaoshiti areas. Thus, further research may be focused on a contrastive study of the Moxi and Gaoshiti areas based on the practical well production effects and palaeogeography restoration.

## Conclusions

We start this study with a reservoir characteristics analysis, identifying the reservoir types and showing their physical properties and distributions. Then, we focus on the sedimentary influence on reservoir development to determine the effects of depositional sequences and sedimentary facies on creating favourable reservoirs of the Deng IV Member in the Gaoshiti-Moxi area of the Sichuan Basin by using data from seismic profiles, cores, thin sections, and well logs. The conclusions are summarized as follows:

1. Fracture-cavity, pore-cavity and pore reservoirs are identified as three main types of reservoirs, with porosities of 1–5%, 2–4%, and 1–3%, respectively; the permeabilities are 0.01–1 × 10^–3^ μm^2^, 0.01–1 × 10^–3^ μm^2^, and 0.001–0.1 × 10^–3^ μm^2^, respectively. The reservoir space is genetically mostly secondary, with the most common types being residual intragranular pores, intergranular pores, intercrystalline pores and cavities.
2. The three types of reservoirs are mainly developed vertically in PSS5-PSS7. Regionally, fracture-cavity reservoirs are mainly distributed in well zone MX9-MX13-MX19-MX17-MX18-MX11 in the shape of stipes. Pore-cavity reservoirs occupy large areas in Gaoshiti and northern Moxi and are continuously distributed in well zones GS7 and GS20. Pore reservoirs are mainly distributed in the Moxi area.
3. High-frequency sequence boundaries affect the vertical reservoir distributions. The fracture-cavity type of reservoir is constrained by the top boundaries of PSS7, PSS2, PS17 and PS14; the pore-cavity type of reservoir is constrained by the top boundaries of PSS7, PSS4, PS18 and PS12; and the pore type of reservoir is constrained by the top boundaries of PSS7, PSS6, PSS3, PSS2, PS18, PS17, PS14, and PS12.
4. Pore cavity and pore reservoir distributions are closely related to seismic facies associated with shoals and mound-flat complexes. Most pore-cavity reservoir facies correspond to seismic facies-associated shoals, such as shallow shoals, mound-shoal complexes, and shoal-flat complexes; most pore reservoir facies correspond to seismic facies of mound-flat complexes; and the fracture-cavity reservoir distribution has little connection to seismic facies.

MA1, MA3, MA7, and MA8 are predominant microfacies associations of favourable reservoirs probably developed in PS19 and PS15. MA1, MA3, and MA7 have positive effects on creating favourable reservoirs in PS19, which corresponds to the top of PSS7; MA8 has a positive effect on creating favourable reservoirs in PS15, which corresponds to the top of PSS6.

## Data Availability

All data, models, or code generated or used during the study are available from the corresponding author by request.
